# Theoretical Estimation Based on CT Images and Experiments on the Sound Absorption Coefficient of Foam Materials with Removed Membrane †

**DOI:** 10.3390/ma18040846

**Published:** 2025-02-14

**Authors:** Shuichi Sakamoto, Takamasa Satoh, Kaito Tanabe, Koki Maruyama, Yusei Himori

**Affiliations:** 1 Department of Engineering, Niigata University, Ikarashi 2-no-cho 8050, Nishi-ku, Niigata City 950-2181, Niigata Prefecture, Japan; 2 Fukoku Co., Ltd., 6 Showa Chiyoda-machi, Oura-gun 370-0723, Gunma Prefecture, Japan; takamasa_satoh@fukoku-rubber.co.jp; 3 Graduate School of Science and Technology, Niigata University, Ikarashi 2-no-cho 8050, Nishi-ku, Niigata City 950-2181, Niigata Prefecture, Japan

**Keywords:** foam absorbent, micro-CT scan images, tortuosity, sound absorption coefficient, transfer matrix method

## Abstract

The structure of foam sound absorbers is not strictly regular, and it is difficult to create a geometric model. In this study, a method for estimating the sound absorption properties of foam sound absorbers with the membrane removed was proposed based on computed tomography (CT) scan images: the circumference of the structure and the cross-sectional area of the voids in the foam cross-section were determined from CT scans of foam materials. The propagation constant and characteristic impedance at the voids were obtained by approximating the foam material cross-section as the clearance between two planes, and the transfer matrix method was used to calculate the normal incident sound absorption coefficient. Further, the sound absorption coefficient was theoretically derived using the effective density with the measured tortuosity applied and compared with the experimental value using a two-microphone impedance measuring tube. By extracting the skeletal part of foam materials by using image processing and removing the residual noise in CT images, and then varying the correction factor for the skeleton surface area, the theoretical value of the sound-absorbing foam without a membrane was closer to the measured value.

## 1. Introduction

Recently, with the electrification of mechanical products, high-frequency noise due to magnetostrictive noise from motors caused by inverters has become apparent [1]. In addition, thin, lightweight sound-absorbing materials that can be used in various shapes are required for noise reduction in moving vehicles, such as automobiles [2,3]. Research on porous materials has focused on evaluating and optimizing their acoustic and mechanical properties, with applications such as road surfacing [4,5]. In addition, studies on recycled porous materials, like polyurethane foams, have highlighted their potential as sound-absorbing materials [6,7].

In this study, we focused on polyurethane foam, which is a porous sound-absorbing material. Polyurethane foams are classified according to their hardness as hard, semi-hard, or soft, and each type has different application areas and properties. Polyurethane foams are used in car dashboards [8] and seats [9], and research [10] has been conducted to recycle polyurethane foam waste from the textile industry. The polyurethane foams used in these applications are soft foams manufactured by mixing a foaming agent. The cell size and membrane proportion vary depending on the manufacturing technique [11,12].

The properties of foam sound-absorbing materials vary greatly depending on the porosity and cell size [12]. Observation of foam sound-absorbing materials via electron microscopy reveals the presence of a membrane on the foam material framework. The membranes of foam materials have not been described in previous studies on foam sound-absorbing materials [13,14]. Meanwhile, experimental studies on the presence or absence of membranes in foam materials have reported that the influence of membranes on the sound absorption coefficient is significant [15,16]. The Kelvin model is a well-known, homogeneous mathematical model for foam materials. However, real foam materials exhibit irregular structures, as revealed by micrographs and CT images. The structure of foam sound absorbers is not strictly regular, and it is difficult to create a geometric model.

Therefore, we theoretically estimate the acoustic properties of foam sound absorbers. As a basic study, a preliminary study was conducted on a sample with its membrane removed to investigate the sound absorption properties of foam sound absorbers in the absence of membranes [17].

In this study, a method for estimating the sound absorption properties of foam sound absorbers with the membrane removed was proposed based on computed tomography (CT) scan images: the circumference of the structure and the cross-sectional area of the voids in the foam cross-section were determined from CT scans of foam materials. The propagation constant and characteristic impedance at the voids were obtained by approximating the foam material cross-section as the clearance between two planes, and the transfer matrix method was used to calculate the normal incident sound absorption coefficient. Further, the sound absorption coefficient was theoretically derived using the effective density with the measured tortuosity applied and compared with the experimental value using a two-microphone impedance measuring tube.

This method for estimating the sound absorption coefficient using CT scan images has yielded positive results for other materials, such as randomly filled granular materials [18], rice and buckwheat husks [19], and rice straw [20]. This study explores the potential for developing image-based models using tomographic images of foam materials, as opposed to the mathematical models typically used for grid structures [21] and frame structures [16] in previous studies. This study is expected to be useful for developing foam sound absorbers based on simulation.

## 2. Sample and Measuring Apparatus

### 2.1. Sample Used for Measurements

A porous foam sound-absorbing material was used in this study. The material was L-25 manufactured by Toyo Quality One (Kawagoe, Saitama, Japan), and samples with a 29 mm diameter and 10 mm height were prepared.

**Figure 1:**
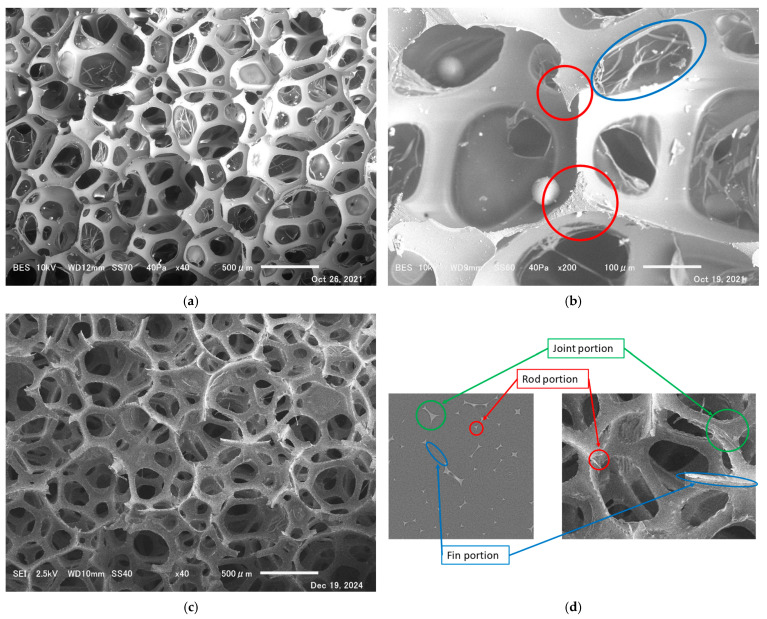
Magnified electron micrograph of the foam sound-absorbing material and the names of its parts (JEOL Ltd. JSM-6010PLUS/LA): (**a**) structure of the foam material and membrane presence, (**b**) membrane (blue circle) and cross-section of the rod portion (red circles), (**c**) image of the foam material with the membrane removed, and (**d**) names of the parts of the framework.

A scanning electron microscope (SEM; JEOL JSM-6010PLUS/LA, Tokyo, Japan) confirmed the structures and films (Figure 1a,b). According to a previous study [16], the percentage of grids with complete membranes was 28% of the total. Figure 1a,b show different magnification scales. In Figure 1b, the areas circled in red and blue are the rod cross-section and membrane, respectively.

Notably, membranes present in sound-absorbing foam materials are as thin as 1.5 ± 0.25 µm [12] and difficult to completely image with the resolution of the micro–X-ray CT used (3.9 µm). Therefore, a comparison was made between the experimental and theoretical values based on CT images for the foam materials with the membranes removed. Foam materials without membranes are known for usage in microphone wind screens and water absorption; however, their sound absorption performance is very poor. As this research concerned the fundamentals of sound-absorbing foams, it dealt with the foam skeleton of sound-absorbing foams. The technique used to remove membranes from foam sound-absorbing materials was rubbing the foam material dozens of times in water, thereby using hydraulic pressure to break the membranes.

Figure 1c shows an SEM image of a foam sound absorber with its membrane removed, at the same magnification as in Figure 1a. Figure 1c shows that the membrane was almost completely removed.

The names of the parts of the foam sound absorber framework are shown in Figure 1d. The rod-like part (like a triangular pillar) is called the rod (portion), the connecting part between the rods is called the joint (portion), and the thin-walled part around the rod or joint that connects to the membrane is called the fin portion.

**Figure 2:**
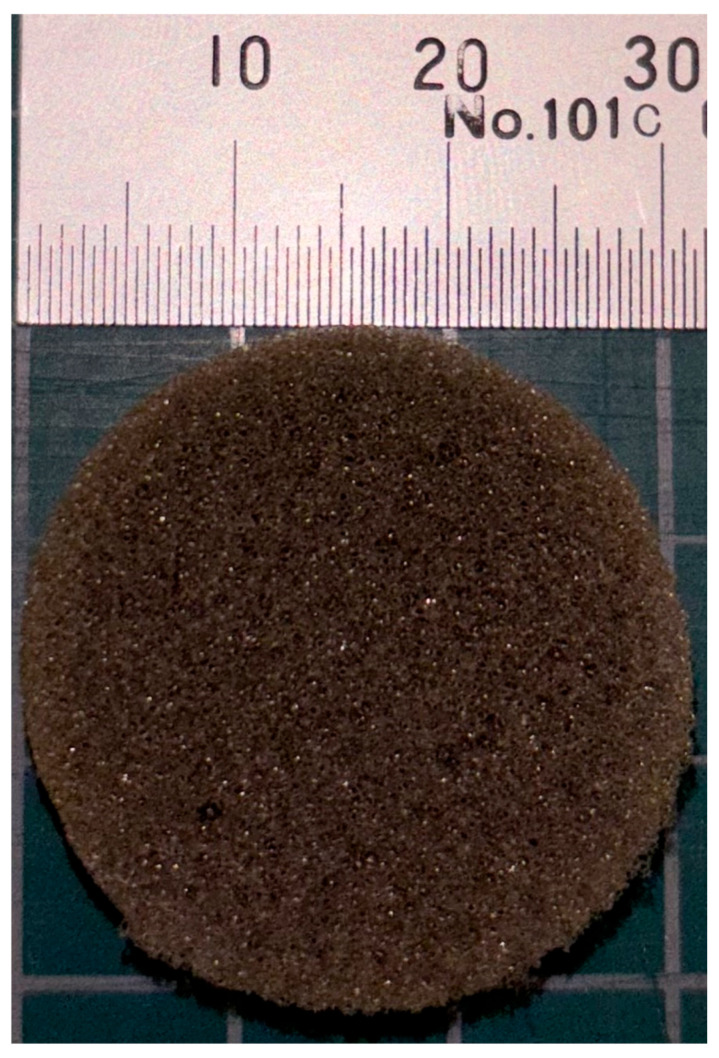
Sample used for impedance tube measurements.

Figure 2 shows a photograph of the sample used to measure the sound absorption coefficient.

### 2.2. Equipment for Measuring the Sound Absorption Coefficient

**Figure 3:**
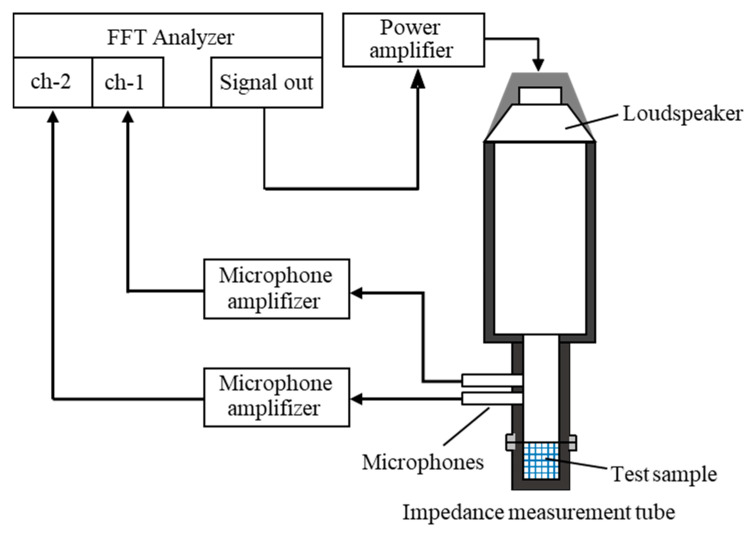
Schematic of two-microphone impedance tube used for absorption coefficient measurement.

A two-microphone acoustic impedance tube (Type 4206 from Brüel & Kjær, Nærum, Denmark) as shown in Figure 3 was used to measure the normal incident sound absorption coefficient. The transfer function between the sound pressure signals from the two microphones was measured using an FFT analyzer. This transfer function was then used to calculate the sound absorption coefficient at normal incidence, following the guidelines of ISO 10534-2 [22]. The sample used in this study did not have a high sound absorption coefficient in the low-frequency range; thus, an impedance tube with an inner diameter of 29 mm was used.

### 2.3. Methods and Results of Measuring Tortuosity

When a material’s internal geometry is complex, sound waves pass along a path longer than the material’s length in the travel direction. Tortuosity is a parameter that expresses the complexity of sound waves as they pass through a material. In this study, the tortuosity of foam sound absorbers was measured using ultrasonic sensors.

**Figure 4:**
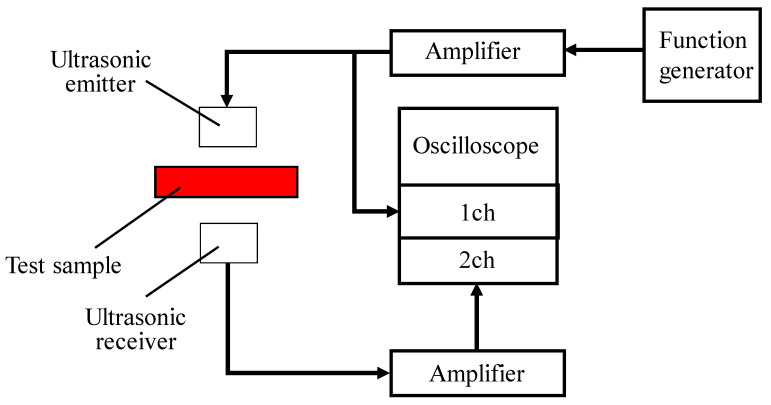
Schematic of tortuosity measurement.

**Figure 5:**
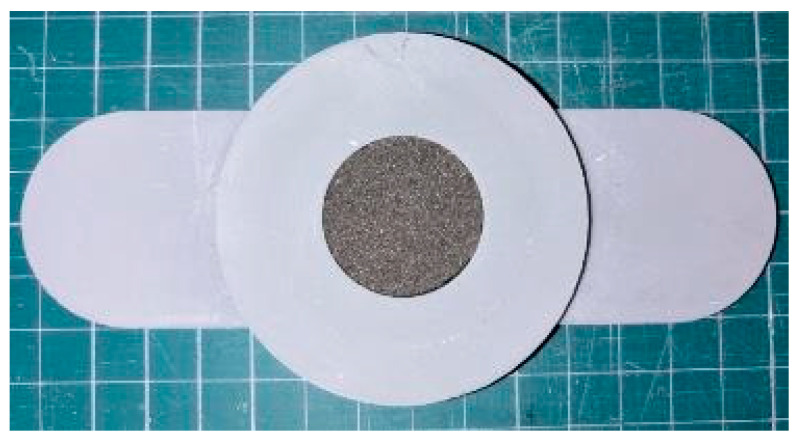
Test sample for tortuosity measurement with a foam sound absorber.

A schematic of the tortuosity measurement system is shown in Figure 4. A sample holder was fabricated using light-curing resin with a Form2 3D printer (manufactured by Formlabs, Somerville, MA, USA) to secure the foam material. The sample holder used for measuring the tortuosity and a photograph of the sample are shown in Figure 5. The same method previously reported [18,19,23] was used to derive the tortuosity.

**Figure 6:**
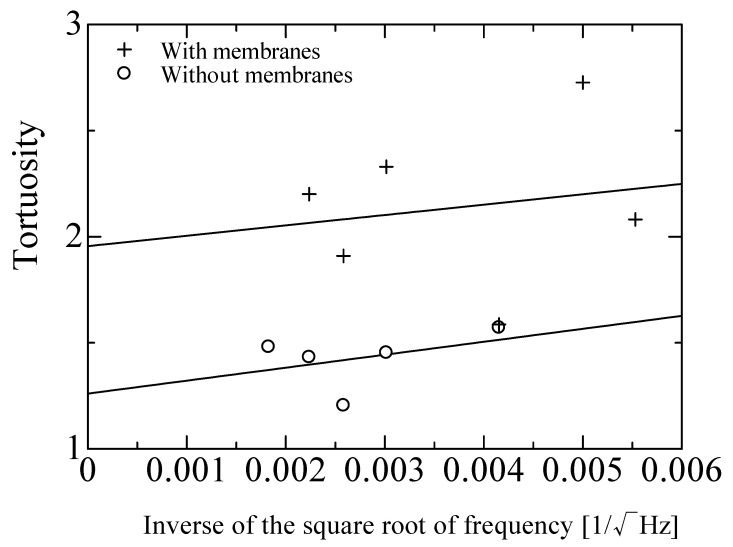
Tortuosity measurement results (foam sound absorber with or without membrane).

The tortuosity measurement results are shown in Figure 6. Ultrasonic sensors with central frequencies of 58, 110, 150, 200, and 300 kHz were used. In Figure 6, the horizontal axis represents the inverse of the square root of the frequency used for measurement, while the vertical axis plots the tortuosity for each frequency, as determined by the measurements. The intercept of the least-squares line in Figure 6 gives a tortuosity of *α_∞_* = 1.26 for the sound-absorbing foam sample without a membrane. The result for the foam sound absorber before the membrane was removed is also shown in Figure 6 for reference, where the tortuosity was determined to be *α_∞_* = 1.95.

## 3. Theoretical Analysis

### 3.1. CT Scan Tomograms

**Figure 7:**
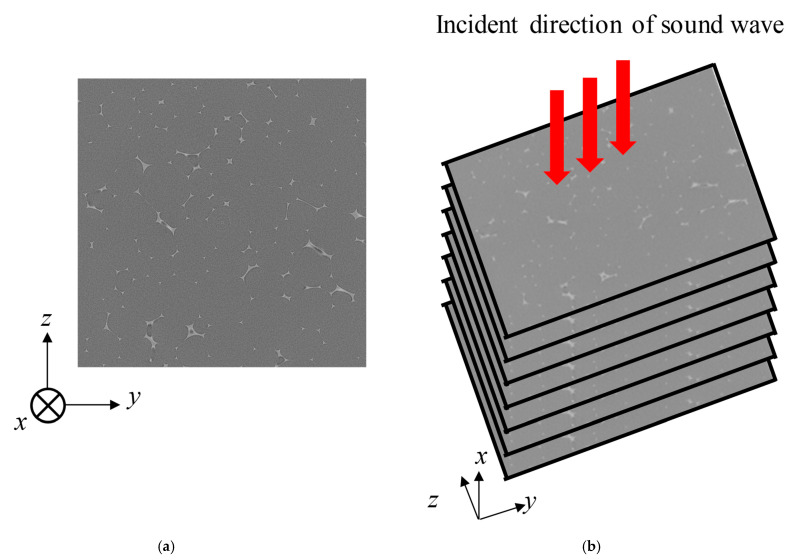
Cross-sectional image of foam sound absorber: (**a**) typical cross-sectional image of an arbitrary point in the *x*-direction without membrane and (**b**) schematic of analysis units.

An example of a CT scan tomogram of a foam material without a membrane is shown in Figure 7a. The equipment used was an MCT225 Metrology CT from NIKON Corp. (Tokyo, Japan).

As shown schematically in Figure 7b, the tomograms were taken in the *y*-*z* plane, which is perpendicular to the sound wave incidence direction (*x*-direction). The area of the images is a square with a side of approximately 4.0 mm. The number of images used for the theoretical analysis was 1,026. Therefore, the pitch of the images in the *x*-direction is approximately 3.9 µm. And the resolution (pixel size) of the image is 3.9 µm square.

### 3.2. CT Images and Image Processing

The internal structure of foam materials is complex (Figure 1a), making it difficult to create analogous mathematical models. Therefore, the sound absorption coefficient was theoretically estimated from the actual structure of foam materials by layering the cross-sectional images using CT.

The CT images were binarized to determine the cross-sectional area of voids. The CT images used were in 8-bit (256 grayscale). They were converted into black-and-white binarized images using a set “threshold” as a boundary. An under- or over-threshold can increase noise in a binarized image and cause the loss of details (e.g., fin portions) observed in the original CT image. Thus, the original CT and binarized images were compared, and the threshold was varied so that the fin portion could be extracted and denoised appropriately.

The amount of noise and the extraction performance of the fin portion differ depending on the spatial resolution of the CT scanner used and the range of the acquisition. Therefore, noise reduction and extraction of the form skeleton were performed in accordance with the tomographic images of the X-ray CT equipment used in this study.

The processing of the tomograms taken by the NIKON Corp. MCT225 Metrology CT, as shown in Figure 7a, is described. The tomograms contain noise. Therefore, if binarization is performed before denoising, excessive noise will remain if a threshold value that allows the fin portion to be extracted is selected. Thus, a general median filter was used for image noise removal with a kernel size of 3.

**Figure 8:**
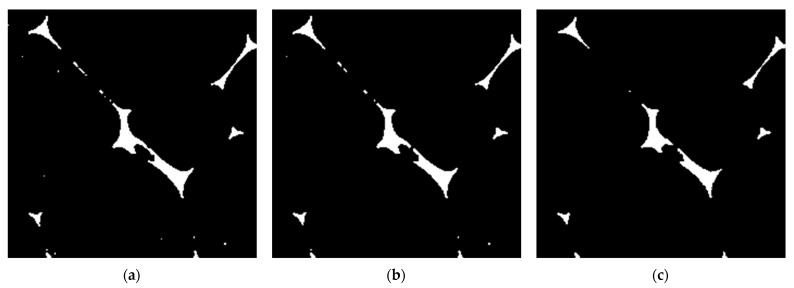
Part of binarized images in Figure 7a scanned by MCT225 Metrology CT (enlarged image of an area of approximately 0.90 × 0.90 mm^2^) with thresholds of (**a**) 0.499, (**b**) 0.500, and (**c**) 0.520.

Figure 8a–c show the binarization results of the X-ray CT scan images after the preliminary processing. The threshold values in Figure 8a–c differ. The cross-sectional area of the voids is determined by the number of pixels in the area identified as black in the images [19].

The Canny edge detection method [24] was applied to the denoised binarized images for edge extraction. This helped sharpen the structural outline and obtain a circumference closer to the actual structure. This gave the circumference of the skeletal part [19].

### 3.3. Approximation of Void to Clearance Between Two Planes

**Figure 9:**
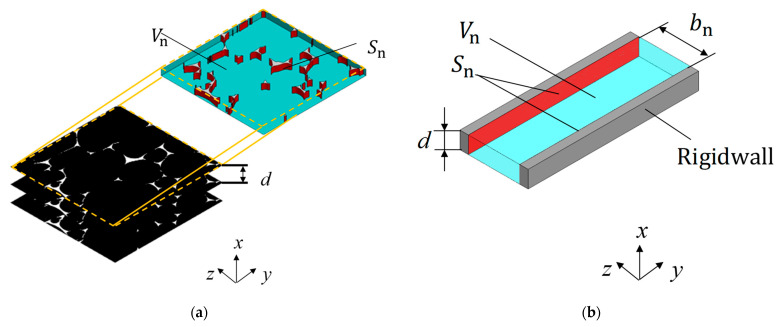
Foam surface area and clearance volume: (**a**) cross-sectional image of an arbitrary point in the *x*-direction and (**b**) two-plane approximation.

**Figure 10:**
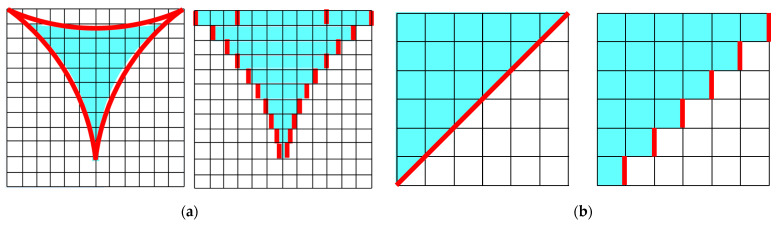
Cross-sectional view of the true and divided shapes of foam frame: (**a**) inside part of three circumscribed circles and (**b**) inclined flat surface.

This section describes the approximation of a void to the clearance between the two planes. The cross-sectional area and circumference of voids were obtained from the binarized and edge-extracted images to approximate the clearance between the two planes. Figure 9a,b show the geometry before and after the approximation to a clearance between two planes, respectively, which are calculated using the cross-sectional area and circumference of the gap obtained in Section 3.2. Multiplying the cross-sectional area of the voids by the pitch d between the images yields the volume of the voids, *V_n_*, shown in Figure 9a. Similarly, multiplying the total circumference of the cross-section by the pitch *d* yields the surface area of the skeletal part, *S_n_*, in Figure 9a. The clearance thickness *b_n_* between the two planes is determined for each image shown in Figure 9b using the relationship between the volume *V_n_* and the surface area *S_n_*, as described in Equation (1):

(1) $$b_{n}=\frac{2V_{n}}{S_{n}\times F}$$

where *F* is a correction factor to account for the true surface area and represents the ratio of the real surface area to *S_n_*. For example, if the foam material skeleton is assumed to be the area surrounded by three circumscribed circles (Figure 10a), the correction factor *F* for obtaining the true surface area is π/2 ≅ 1.57. If the skeleton is assumed to be a flat plate inclined at 45° in the *x*-direction (Figure 10b), the correction factor *F* is √2 ≅ 1.414.

### 3.4. Propagation Constant and Characteristic Impedance

The propagation constant and characteristic impedance are derived by considering the attenuation of sound waves in the voids for which two-plane approximation was performed. Tjideman [25] and Stinson [26] studied circular tubes, Stinson [27] studied equilateral triangle tubes, and Beltman [28] studied rectangular tubes. Moreover, Allard [29] considered the degree of tortuosity in his analysis. The methods of Stinson [27] and Allard [29] were applied in this study.

**Figure 11:**
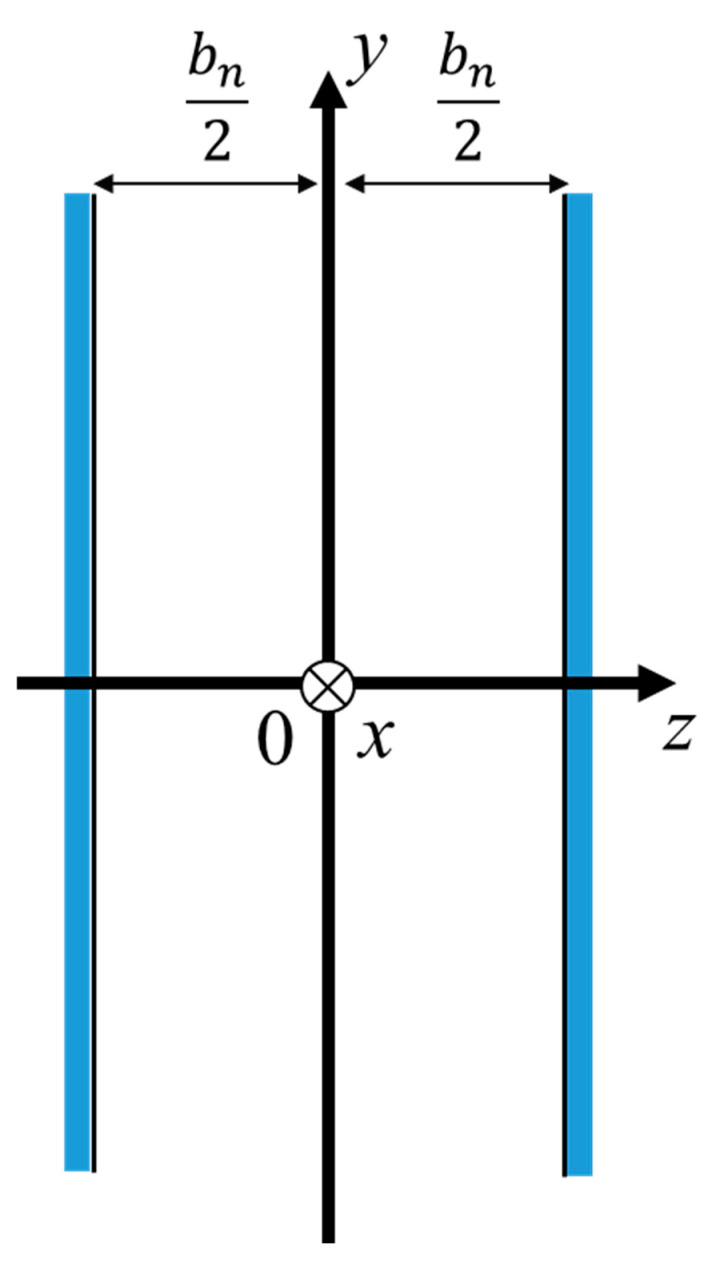
Cartesian coordinate system for parallel space between two planes.

The Cartesian coordinate system is considered (Figure 11), and a three-dimensional analysis is performed using the Navier–Stokes equations, gas equation of state, continuity equation, energy equation, and dissipative function representing heat transfer to derive the effective density *ρ_s_* and compression ratio *C_s_*, as shown in Equations (2) and (3), respectively:

(2) $$\rho_{s}=\rho_{0}{\left[1-\frac{\tanh\left(\sqrt{j}\lambda_{s}\right)}{\sqrt{j}\lambda_{s}}\right]}^{-1},\;\lambda_{s}=\frac{b_{n}}{2}\sqrt{\frac{\omega \rho_{0}}{\eta }}$$

(3) $$C_{s}=\left(\frac{1}{\kappa P_{0}}\right)\left\{1+\left(\kappa -1\right)\left[\frac{\tanh\left(\sqrt{jN_{pr}}\lambda_{s}\right)}{\sqrt{jN_{pr}}\lambda_{s}}\right]\right\}$$

These values were derived from the work of Stinson [26] and Allard [29], where *ρ_0_* denotes the air density, *λ_s_* denotes the mediator variable, *b_n_* denotes the thickness of the air clearance between the two planes, *ω* denotes the angular frequency, *η* denotes the air viscosity, *κ* denotes the specific heat ratio of air, *P_0_* denotes the atmospheric pressure, and *N_pr_* denotes the Prandtl number.

Using the effective density *ρ_s_* and compressibility *C_s_* outlined above, the propagation constant *γ* and characteristic impedance *Z_c_* are expressed by the following equations, based on the definitions provided by Stinson [26,27] and others:

(4) $$\gamma =j\omega \sqrt{\rho_{s}C_{s}}$$

(5) $$Z_{c}=\sqrt{\frac{\rho_{s}}{C_{s}}}$$

By the effective density multiplied by the tortuosity, the propagation constant and characteristic impedance considering the tortuosity can be obtained [29]. Therefore, the propagation constant and characteristic impedance when considering the tortuosity *α_∞_* can be expressed as follows:

(6) $$\gamma =j\omega \sqrt{{\alpha_{\infty }\rho }_{s}C_{s}}$$

(7) $$Z_{c}=\sqrt{\frac{\alpha_{\infty }\rho_{s}}{C_{s}}}$$

### 3.5. Transfer Matrices and Their Connections

**Figure 12:**
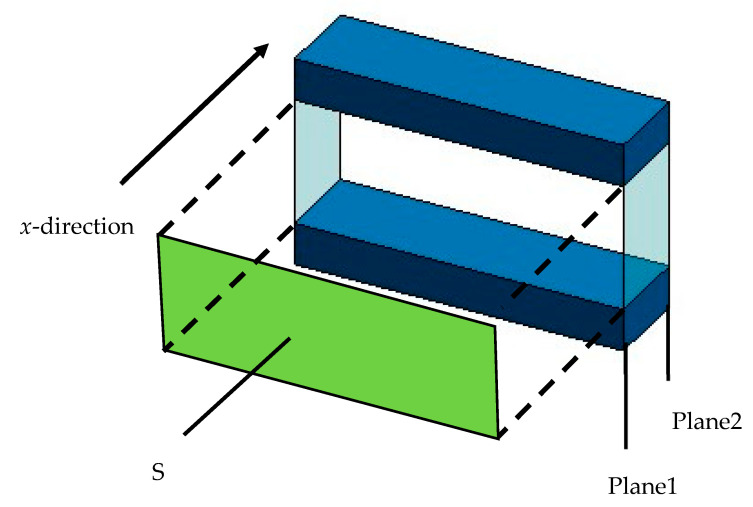
Sound incident area, incident plane, and transmission plane of approximated clearance between the two planes.

The clearance between the two planes was analyzed using the transfer matrix method for sound pressure and volume velocity based on a one-dimensional wave equation. Figure 12 shows a schematic of one element in the *x*-direction for the clearance between the two planes shown in Figure 11.

Using the cross-sectional area *S* of the clearance, pitch *d* per layer, characteristic impedance *Z_c_*, and propagation constant *γ*, the transfer matrix *T* and four-terminal constants *A*~*D* of the acoustic tube element can be expressed as follows:

(8) $$T=\left[\begin{array}{cc} \cosh\left(\gamma d\right) & \frac{Z_{c}}{S}sinh\left(\gamma d\right)\\ \frac{S}{Z_{c}}sinh\left(\gamma d\right) & \cosh\left(\gamma d\right) \end{array}\right]=\left[\begin{array}{cc} A & B\\ C & D \end{array}\right].$$

Plane 1 is the sound wave incidence plane, and Plane 2 is the sound wave transmission plane; if the sound pressure and particle velocity in Plane 1 are *p_1_* and *u_1_*, respectively, and the sound pressure and particle velocity in Plane 2 are *p_2_* and *u_2_*, respectively, the transfer matrix is expressed as follows:

(9) $$\left[\begin{array}{c} p_{1}\\ Su_{1} \end{array}\right]=\left[\begin{array}{cc} A & B\\ C & D \end{array}\right]\left[\begin{array}{c} p_{2}\\ Su_{2} \end{array}\right].$$

By applying Equation (9) to the clearance between the two planes derived by image processing, the transfer matrix for each segmented element is derived.

**Figure 13:**
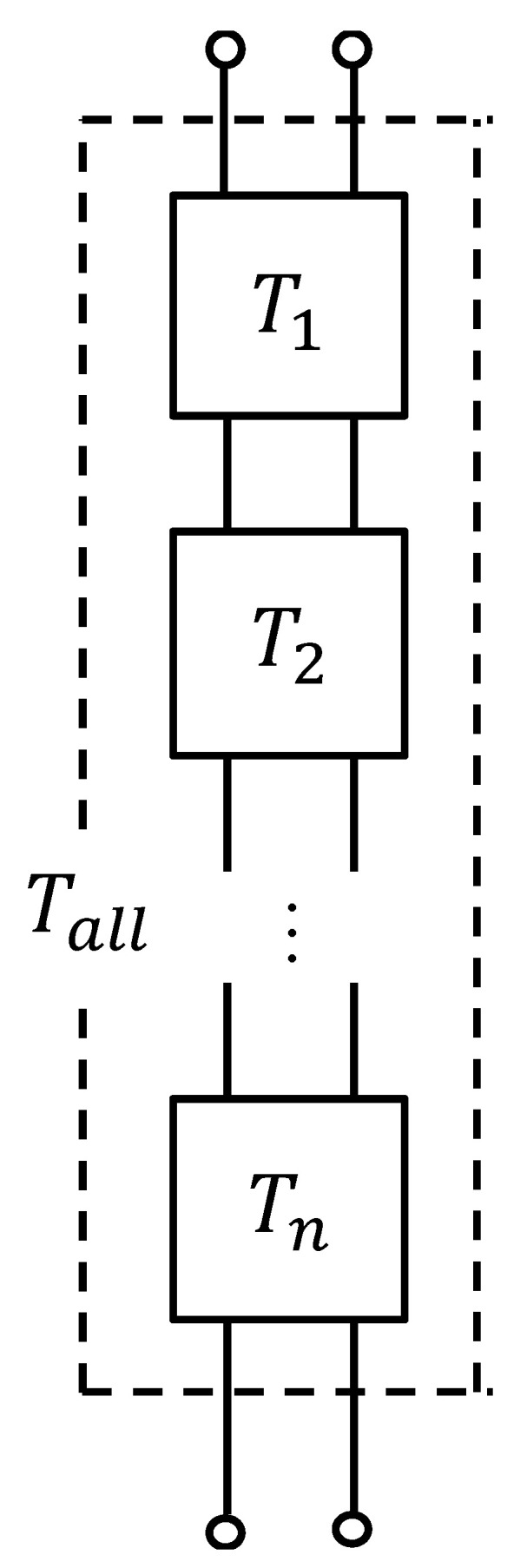
Cascade connecting *T_all_*.

Based on the equivalent circuit shown in Figure 13, the transfer matrix *T_all_* for the entire sample is derived by connecting the transfer matrices of each successive segment in the *x*-axis direction in the cascade. The number of transfer matrices connected in the cascade is equal to the number of images, i.e., the image pitch multiplied by the number of images gives the height of each sample.

### 3.6. Derivation of Normal Incident Sound Absorption Coefficient

The sound absorption coefficient is derived from the transfer matrix *T_all_* obtained in Section 3.5. Applying the acoustic tube of Figure 11 to the actual sample, Plane 2 is a rigid wall. Therefore, because the particle velocity *u_2_* = 0, Equation (9) can be transformed as in Equation (10) to obtain Equation (11).

(10) $$\left[\begin{array}{c} p_{1}\\ Su_{1} \end{array}\right]=\left[\begin{array}{cc} A & B\\ C & D \end{array}\right]\left[\begin{array}{c} p_{2}\\ 0 \end{array}\right]$$

(11) $$\left[\begin{array}{c} p_{1}\\ Su_{1} \end{array}\right]=\left[\begin{array}{c} Ap_{2}\\ Cp_{2} \end{array}\right]$$

According to Equation (10), the specific acoustic impedance *Z*_1_ looking into the interior of the sample from Plane 1 can be expressed as follows:

(12) $$Z_{1}=\frac{p_{1}}{u_{1}}=\frac{p_{1}}{{Su}_{1}}S=\frac{A}{C}S.$$

The relationship between the specific acoustic impedance *Z*_0_ and reflectance *R* is expressed as follows:

(13) $$R=\frac{Z_{1}-\rho_{0}c_{0}}{Z_{1}+\rho_{0}c_{0}}$$

The following relationship between the sound absorption coefficient and reflectance and Equation (13) gives the theoretical value of a sample’s normal incident sound absorption coefficient *α*:

(14) $$\alpha =1-{\left|R\right|}^{2}$$

## 4. Comparison of Calculated and Measured Results

### 4.1. Theoretical Values Obtained by CT Images with Varying Binarization Thresholds

**Figure 14:**
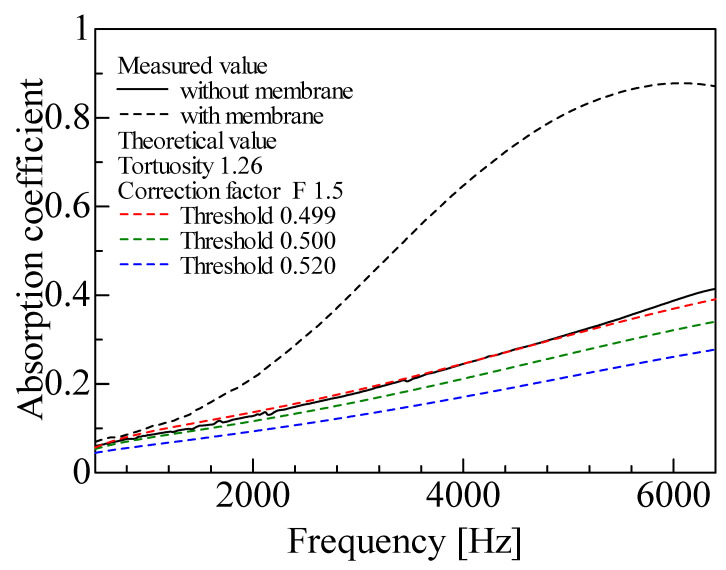
Comparison of experimental and theoretical results using images scanned by MCT225 Metrology CT: theoretical values due to changes in threshold.

The theoretical values obtained based on the CT images with varying binarization thresholds (Figure 8) are compared with the measured values. The sound absorption coefficient based on CT images with varying binarization thresholds is shown in Figure 14, together with the measured values.

For reference, Figure 14 also shows the experimental results for a foam sound absorber with the membrane present. The sound absorption coefficient is higher in all frequency ranges when the membrane is present than after the membrane removal.

Figure 14 shows changes in the sound absorption coefficient due to changes in the threshold value. As shown in Figure 8, this is thought to be due to differences in the amount of residual noise and recognition of the form skeleton cross-section caused by changes in the threshold value, resulting in changes in the sound absorption coefficient. As the threshold is decreased, the fin portion can be extracted, but the noise increases in the entire binarized image. Meanwhile, if the noise reduction effect is strengthened by increasing the filter or threshold value, the fin portion is also removed as noise. This problem is attributable to the lack of image resolution for the smallest foam material details, which makes it difficult to distinguish between the noise and fin portions.

### 4.2. Sensitivity Analysis of the Correction Factor for Theoretical Values Calculated from CT Images

For the reasons given in Section 3.3, a sensitivity analysis was performed on the correction factor *F* to approximate the true surface area of the skeleton. That is, the threshold is fixed at a representative value, and the theoretical values are compared with the measured values while varying the correction factor *F*.

**Figure 15:**
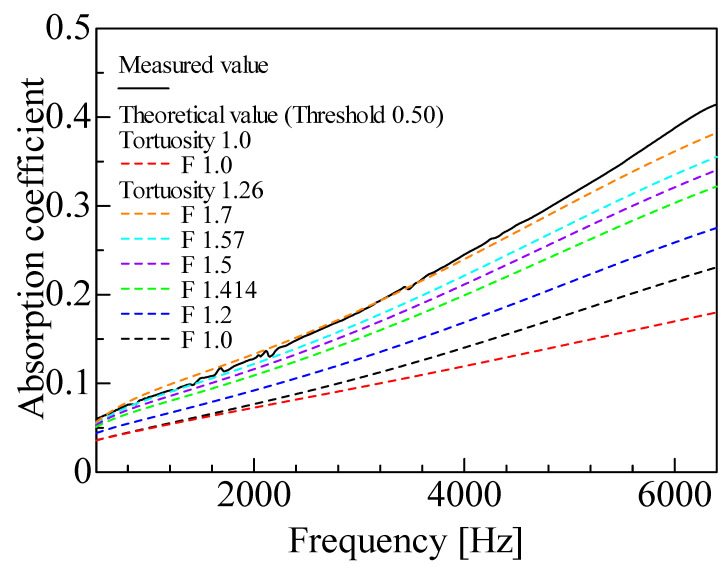
Comparison of experimental and theoretical results using images scanned by MCT225 Metrology CT: theoretical values due to changes in the correction factor.

Figure 15 shows the sound absorption coefficient for varying the correction factor *F* in CT images. For reference, the theoretical values are shown when both the tortuosity and correction factor *F* are not considered. In this case, the lowest sound absorption coefficient is shown in Figure 15.

The sound absorption coefficient increases with the correction factor *F* because the surface area reflected in the analysis increases with the correction factor *F*.

According to Section 3.3 and previous studies [19], a correction factor *F* of approximately 1.4–1.6 is considered suitable. However, in Figure 14, the best agreement between the theoretical and experimental values is found when the correction factor *F* is approximately 1.7. In the foam materials with removed membranes, it is considered that the membranes are not completely removed but remain partially in place. Consequently, if the remaining membranes and other details below the CT imaging resolution are not fully captured, as in the CT images in this study, the calculated sound absorption coefficient is expected to be smaller than the experimental value. A further discussion of the differences between experimental and theoretical values is presented in Section 4.3.

### 4.3. Discussion of Differences Between Measured and Theoretical Values

Based on the above results, the differences between the measured and theoretical values are discussed.

The first cause is the image processing complexity: during the preparation stage of the CT scan image binarization, an attempt was made to apply Otsu’s binarization [30], a typical method for determining threshold binarization values, but the fin portion was excessively removed. Therefore, a threshold was determined to an appropriate value while visually observing an image, and the image was processed to improve the recognition of the fin and other parts. As shown above, the recognition of fin portions is sensitive to the threshold value, and whether the fin surface area is close to the true value or not influences the difference from the measured value.

The second cause is that the samples, used for the CT scanning and sound absorption coefficient measurement, were cut from the same piece of material; however, they are strictly portions of different locations.

The third cause is the lack of detail extraction. In Figure 14, the theoretical value for the 0.499 threshold best agrees with the experimental value. However, examining the image after binarization, the 0.500 threshold in Figure 8b is the most appropriate due to the combination of noise removal and detail extraction. The shortfall of the theoretical value from the binarized image with the 0.500 threshold compared with the experimental value is attributable to the lack of detail extraction. Here, the fact that the 0.499 threshold case is closest to the experimental value is assumed to be because the excess residual noise compensates for the lack of detail extraction.

## 5. Conclusions

The normal incident sound absorption coefficient was theoretically derived using CT images of foam sound absorbers with the membranes removed. The circumference of the foam sound absorber framework and the cross-sectional area of voids were determined by CT image processing, and the propagation constant and characteristic impedance were derived. Tortuosity, which indicates the complexity of the sound wave propagation path, was measured and introduced into the theoretical analysis. The measured and theoretical values of the normal incident sound absorption coefficient were compared and evaluated.

Theoretical analysis was performed based on CT images of foam sound absorbers for which geometrical modeling is difficult due to the lack of strict regularity in the structure, and the sound absorption coefficient was theoretically derived. By varying the correction factor for the skeleton surface area, the theoretical value of the sound-absorbing foam without a membrane was closer to the measured value. Therefore, the theoretical estimation of the sound absorption coefficient using CT images can be useful.

## Data Availability

The original contributions presented in this study are included in the article. Further inquiries can be directed to the corresponding author.
